# The Structure and Flexural Properties of *Typha* Leaves

**DOI:** 10.1155/2017/1249870

**Published:** 2017-10-15

**Authors:** Jingjing Liu, Zhihui Zhang, Zhenglei Yu, Yunhong Liang, Xiujuan Li, Luquan Ren

**Affiliations:** ^1^Key Laboratory of Bionic Engineering, Ministry of Education, Jilin University, Changchun 130025, China; ^2^State Key Laboratory of Automotive Simulation and Control, Jilin University, Changchun 130025, China

## Abstract

The *Typha* leaf has a structure of lightweight cantilever beam, exhibiting excellent mechanical properties with low density. Especially, the leaf blade evolved high strength and low density with high porosity. In this paper, the structure of *Typha* leaf was characterized by microcomputed tomography (Micro-CT) and scanning electron microscopy (SEM), and the relationship with flexural properties was analyzed. The three-point bending test was performed on leaves to examine flexural properties, which indicated that the flexural properties vary from the base to the apex in gradient. The cross-sectional geometry shape of the leaf blade presented a strong influence on the optimized flexural stiffness. The load carrying capacity of the leaf depended on the development level of the epidermal tissue, the vascular bundle, the mechanical tissue, and the geometric properties. The investigation can be the basis for lightweight structure design and the application in the bionic engineering field.

## 1. Introduction

*Typha glauca*, also named water candle, usually grows in shallow water marshes as marsh perennial herbaceous plants. Owing to the low-cost and large surface area that are raw potential for fabricating the activated carbon adsorbent [1, 2]. *Typha* plants are widely used in constructing wetlands for ecological restoration [3] and sewage treatment [4–6]. In addition, the *Typha* is also the raw material for heat preservation [7], weaving, and paper [8] due to its fiber length, toughness, and heat preservation performance.

More importantly, the *Typha* leaf can be considered as a lightweight cantilever beam with impressive length and a large slenderness ratio. This is because the normal vertical growth of *Typha* leaves is ensured by excellent mechanical properties in spite of the forces of gravity, wind, and rain. In previous studies, the structure of *Typha* leaves was mainly referenced on the anatomical classification of species and genetic variation [9–12]. Recently, the composite material researches regarding the *Typha* plant fiber have been started [13–15] and gradually become a novel highlight. Natural fibers are often used as reinforcement for composite materials to increase specific tensile strength and energy absorption. As a biomaterial, the *Typha* leaf is not homogeneous and its structural response is determined by several factors including the cell's material, the arrangement of the tissues, the way in which the fibers are assembled, and the degree of interaction between them. Moreover, the leaf of *Typha* has a sandwich-type structure and the continuous leaf-handed twist to prevent buckling by natural conditions and external force of storms [16]. The very fine fibre cables in the air compartments of the *Typha* leaf that are strong under tension form a tensegrity structure, which creates multiple load paths through which stresses are redistributed throughout the 1–3 m tall upright leaves [17]. Until recent years, the mechanical properties of the *Typha* leaf were examined by uniaxial tension and three-point bending tests, and the results showed the maximum load, elastic modulus, and stiffness of blade which presents gradient variations in the height direction [18, 19]. Although the aforementioned works have illustrated the mechanical behavior of the *Typha* leaf in a way, but the relationship between structure and mechanical properties of the *Typha* leaf is still not clear.

In this paper, Micro-CT and SEM were used to observe the three-dimensional macroscopic morphology and microstructure of the *Typha* leaf. The cross sections of the leaf from the base to apex were recorded by a digital camera. Then, the effect of cross-sectional geometry shape on the flexural stiffness was studied by using the method of shape transformers. Three-point bending test was performed to examine flexural properties. And the maximal break load, flexural strength, flexural modulus, and the surface elongation at break of leaves were determined. The relationship of structure and mechanical properties of *Typha* leaves were analyzed to provide natural inspiration in light weight designing and applications.

## 2. Materials and Methods

### 2.1. Materials

The fresh and mature *Typha* leaves used in this study were collected in random from Nanhu Park in Changchun, China. The samples were wrapped in preservative films and stored in a refrigerator at 4°C.

### 2.2. Three-Dimensional Macroscopic Morphology Observation

A Skyscan 1172 X-ray desktop microtomograph imaging (Skyscan 2005, Skyscan N.V., Vluchtenburgstraat, Aartselaar, Belgium) was employed for external as well as internal structures of sample and calculated porosity characterization. CT sample was acquired from the middle of the fresh *Typha* blade with the length of 12 mm. The sample was scanned with a voltage of 40 kV, a current of 250 *μ*A, and an exposure time of 230 ms. In order to minimize the Poisson noise, rotation step size of 0.4 combined with random movement and multiple-frame averaging was used during imaging. We chose 9.99 *μ*m pixel size and took 1221 photographs. Then Bruker's CTAn and Bruker's CTVox software was employed to reconstruct 3D cross-sectional structure of the samples by 1221 images.

### 2.3. The Microstructure Observation

*Typha* leaves were cleansed with distilled water and dried before observation. The samples (cross section, coronal section, and longitudinal section) in the base and middle were cut using small forceps from the side of the blade. A 6 nm thick gold film was coated on each sample using 108 vacuum ion coating machine. After that, the base and middle microstructures of the blade were observed by SEM (Model EVO-18, GER). Then, the details of the inner structure of the leaf were obtained.

### 2.4. Shape Transformers and Three-Point Bending Test

The material and the structure of the cross section play important roles in flexural properties of the whole leaf. The effect of the cross-sectional structure on the mechanical properties has been widely studied by the method of shape transformers [20, 21]. With this method, a geometrical quantity of the cross section is normalized by the same geometrical quantity of the surrounding envelope, which is the rectangle defined by the main cross-sectional size. The shape transformer of a geometric quantity, *g*, is defined by

(1) $$\begin{array}{c} \;\psi_{g}=\frac{g}{g_{D}}, \end{array}$$

where *g*_*D*_ is the geometric quantity of the envelope. For example, shape transformers for area and second moment of area about *x*-axis can be defined as

(2) $$\begin{array}{c} \begin{array}{c} \psi_{A}=\frac{A}{A_{D}},\\ \psi_{I_{xx}}=\frac{I_{xx}}{I_{D}}. \end{array} \end{array}$$

Here, we use the Lamé curves to define families of shapes and to formulate the shape transformers [21]. In their implicit form, these curves are given by

(3) $$\begin{array}{c} {\left|\frac{x}{a}\right|}^{n}+{\left|\frac{y}{b}\right|}^{n}=1, \end{array}$$

where *n* can be any rational number, and *a* and *b* are positive real numbers describing the radii of the oval shape.

Three-point bending test was performed on the *Typha* leaves to collect flexural properties by an in situ bending testing machine (Model Mtest50, China), with strain rate of 3.0 mm/min under room temperature. Three typical *Typha* leaves were selected for the bending test. According to the bending degree of the ventral surface, samples were named concave number 1, convex number 2, and flat number 3, respectively. From the base to apex, each leaf was cut into six segments of specimens with about 4 cm length and then subjected to bending test. The force-displacement curves were recorded automatically. Simultaneously, the cross sections of the specimens were monitored by digital camera, and the cross-sectional shape performance has been examined (shown in Table 1). The flexural strength (*σ*), flexural modulus (*E*), and surface elongation at break (*ε*) are calculated by (4), (5), and (6), respectively.

(4) $$\begin{array}{c} Flexural\;strength\;\sigma =\frac{M}{W}, \end{array}$$

(5) $$\begin{array}{c} flexural\;modulus\;E=\frac{{FL}^{3}}{48If}, \end{array}$$

(6) $$\begin{array}{c} elongation\;at\;break\;\varepsilon =\frac{6fH}{L^{2}}, \end{array}$$

where *F* is the maximum load (N), *L* is the range (mm), and *M* is the maximum bending moment and when in the three-point bending test (*M* = *FL*/4), *W* is the section modulus in bending (mm^3^), *H* is the specimen thickness (mm), and *f* indicates the defection (mm).

## 3. Results and Discussion

### 3.1. Three-Dimensional Macroscopic Morphology Observation and Analysis

**Figure 1:**
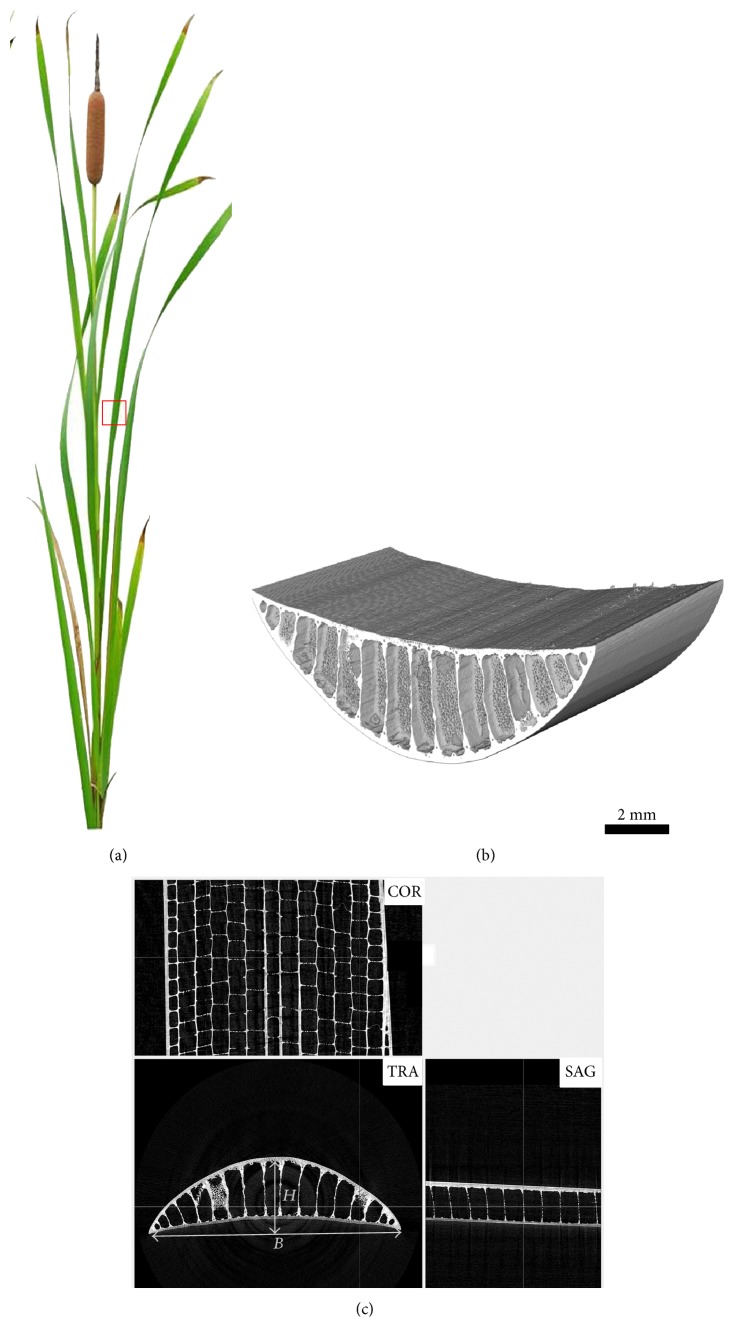
(a) The image of the *Typha* plant. Micro-CT images of a small section (red square part in (a)) in the middle of the *Typha* leaf: (b) 3D structure; (c) three basic orthographic views: transverse section (TRA), coronal section (COR), and sagittal section (SAG). *B* and *H* are the width and thickness of the cross section, respectively.

The *Typha* leaf was studied as shown in Figure 1(a). Synchrotron radiation CT images of a small section in the middle of the *Typha* leaf are shown in Figures 1(b) and 1(c).  Figure 1(b) shows the 3D structure of the leaf. The epidermis, diaphragm, and partition make the leaf to be a lightweight cantilever beam. According to the results from the CT analysis, the volumes of sample and pore space were 4.87 × 10^12^ *μ*m^3^ and 4.68 × 10^12^ *μ*m^3^, respectively. So the porosity (percentage) of this small sample was up to 96%.

Figure 1(c) illustrates three basic orthographic views of the sample. The transverse section (TRA) of the leaf shows a crescent shape. The ventral and dorsal surfaces of the leaf are separated by partitions to form 17 compartments. The aerenchyma compartment is formed in *Typha* leaves through cell lysis in which programmed cell death is involved [22]. The large volume of air-filled compartment allows gas exchange between the aerial and submerged parts [23] and reduces leaf weight, making plants adapt to float in the water. Ventral surface, dorsal surface, and partition consist of many vascular bundles and fibers which play important roles in supporting the whole leaf. Coronal section (COR) possesses a uniform grid structure, which is formed by the staggered arrangement of the horizontal diaphragms in adjacent columns of the compartments. Sagittal section (SAG) indicates that the diaphragms connect to the dorsal and ventral surfaces in the compartment. And all diaphragms are parallel with each other. The parallel diaphragms may contribute to increasing the flexural stiffness of leaves. The width (*B*) and thickness (*H*) of the cross section were used in later geometry parameter measurement.

### 3.2. Microstructure Observation and Analysis

**Figure 2:**
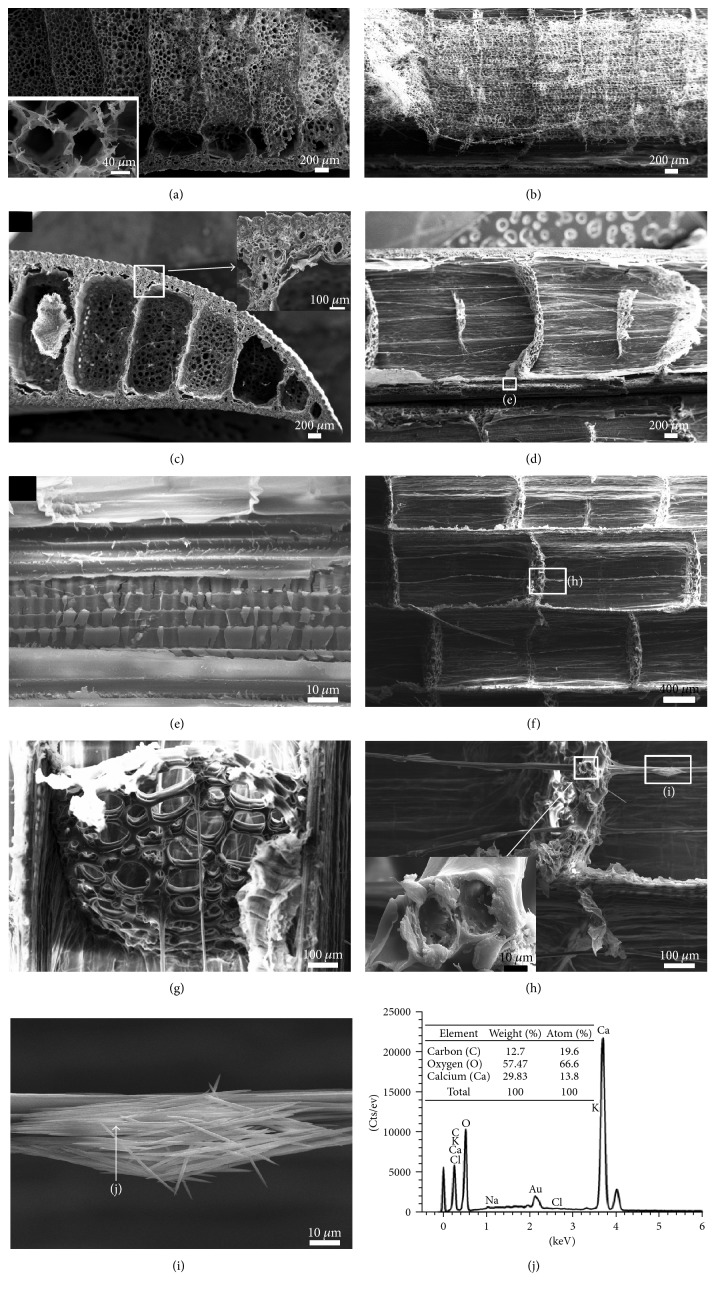
SEM images of the *Typha* leaf blade. (a) Cross section and (b) longitudinal section morphologies of the base of the leaf blade. (c) Cross section and (d) longitudinal section morphologies in the middle of the leaf blade. (e) The magnification of white square part in (d) shows longitudinal section of the vascular bundle. (f) Coronal section in the middle of the leaf. (g) Fiber cables and diaphragms. (h) The aerenchyma tissue of diaphragm and the crystals on the fiber cables amplified by white square part in (f). (i) Raphide crystals. (j) SEM X-ray energy dispersive elemental analysis of raphide crystals.

The morphological characteristics of the base and the middle of the *Typha* leaf are shown in Figure 2. It shows that the leaf contains epidermis, diaphragm, partition, nonlignified fiber cables, and foam tissues. Figures 2(a) and 2(b) show the cross and longitudinal sections of the base of the leaf blade, respectively. Figures 2(c) and 2(d) are the cross and longitudinal sections of the middle of the leaf blade, respectively. It can be found that the middle compartment is filled with foam tissues to form a network at the base of the leaf. However, there is only a small amount of foam tissues existing in the middle of the leaf. In addition, there are more diaphragms at the base than that in the middle acting as a cantilever beam structure. The base of the leaf supports the whole weight of the blade and resists more load than that near the apex during state of bending. Since stiffness depends on core thickness, the extra strength needed in the lower part of the leaf can be achieved simply by thickening the lightweight core [16]. Therefore, the lightweight porous foam tissues and diaphragm on the base of the leaf effectively improve the stability of leaf base and prevent bending failure to a large extent.

*Typha* leaves are rigid with subepidermal vascular and fiber bundles to maintain stability in severe weather. Vascular bundles and fiber bundles distribute in the partitions, dorsal, and ventral surface epidermis along the height direction of the leaf. The vascular bundles are composed of spring fibers (as shown in Figure 2(e)). The continuous vertical partition as an I-beam construction connects the dorsal and ventral surfaces to reduce the stresses of some wind-induced bending.  Figure 2(f) shows that the diaphragms are parallel with each other and perpendicular to partition. In Figure 2(h), the diaphragms composed of aerenchyma tissue which consists of two or three layers of thin-walled stellate cells (Figure 2(g)). The existence of diaphragms in the leaf can prevent lateral shearing, maintain the curvature of the external epidermis, and provide closed box rigidity. In spite of these excellent functions, the diaphragms traversed by very fine fiber cables can also reduce the leaf weight and strengthen the tensegrity structure [17].

Fiber cables traverse the air compartments, as shown in Figure 2(h). The outer surface of the cable has a lot of raphide crystals (Figure 2(i)). The crystals in fiber cables are composed of calcium oxalate which was examined by SEM X-ray energy dispersive elemental analysis [24], as shown in Figure 2(j). The formation of calcium oxalate crystal has an effect on ion balance and osmotic regulation of the leaf. Another major function of calcium oxalate crystals was to provide structural supports or to act as a protective device against foraging animals [25]. In addition, the distribution of calcium oxalate on the fiber surface may contribute to fiber strength and increases the flexural strength of fiber under bending.

### 3.3. The Effect of Cross-Sectional Shape in the Flexural Stiffness

Eighteen cross-sectional geometries of three typical *Typha* leaves were collected and analyzed. As shown in Table 1, the cross section of the leaf varies obviously from crescent to less concave shape, and the leaf is finally biconvex in its distal portion. Near the apex of the sheath, the leaves become separated so that the support between the leaves are weaker, which results in the change of cross-sectional shape. Both the *B* and *H* of the cross-sectional outline decrease from the base to the apex, and the *H* decreases more obviously. The second moment of the area also decreases with the decreasing of the cross-sectional area. That cross-sectional area of the base is 5.87 ~ 6.55 times larger than that at the apex. However, the second moment of the area on the base is 114.64 ~ 152.05 times larger than that at the apex.

**Figure 3:**
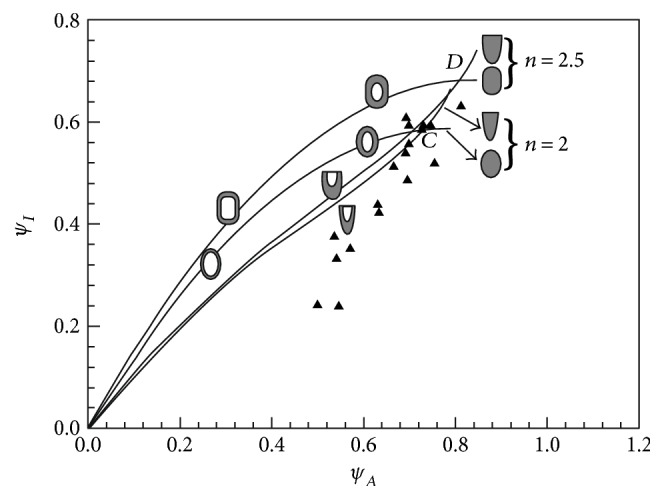
Flexural stiffness curves for cross sections with vertical axis symmetry and flexural stiffness coordinates for leaf specimens (Table 1).

The cross section of a *Typha* leaf has a crescent moon-like shape. To compare cross-sectional shape of the leaf efficiency with that of symmetric and asymmetric superellipses, we selected *n* = 2 and 2.5 in the Lamé curves equation. The ratio *ψ_I_*/*ψ_A_* is a reference of the lightweight efficiency in bending stiffness design of a uniform and isotropic material distribution. The higher the ratio value, the stiffer is the bending and the lighter is the shape. The flexural stiffness has been plotted in Figure 3 and compared with that of the leaf specimens (Table 1).

Figure 3 illustrates the flexural stiffness of both symmetric and asymmetric superellipses with respect to fraction and location of material within the envelope. For pure bending stiffness, cross-sectional size changes have no impact on the flexural stiffness shape properties [21]. The point of each curve represents the bending stiffness of the cross-sectional shape for a given volume and regardless of material. The asymmetric semisuperelliptic shapes are stiffer than the corresponding symmetric concepts when *ψ*_*A*_ > (*ψ*_*A*_)_*C*_ and *ψ*_*A*_ > (*ψ*_*A*_)_*D*__,_ the half-Lamé curves, will make a more economical use of material when they are almost solid. The locations of solid triangle points illustrate that the cross-sectional shapes of *Typha* leaves have a high *ψ_I_/ψ_A_* ratio. Therefore, *Typha* leaves are quite efficient in providing flexural stiffness.

### 3.4. Flexural Properties of the Typha Leaves in Three-Point Bending Test

**Figure 4:**
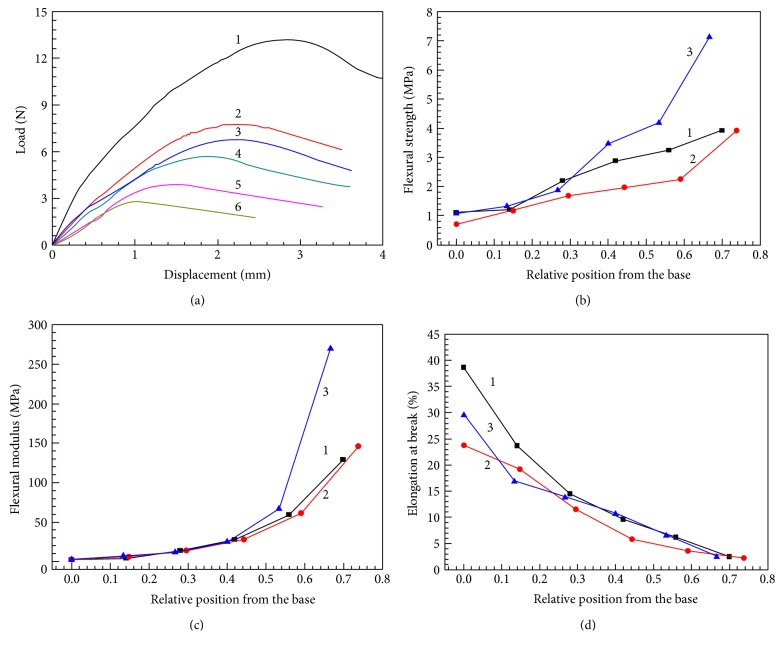
Flexural properties. (a) Typical force-displacement curves of the *Typha* leaf in the three-point bending test, where the numbers 1–6 are the six positions from the base to the apex, respectively. The variations in (b) flexural strength, (c) flexural modulus, and (d) elongation at break of three *Typha* leaves from the base to the apex.

Three-point bending tests were performed to measure the bending properties of three *Typha* leaves, as shown in Figure 4. Six typical load-displacement curves of six different positions along the height direction are plotted in Figure 4(a). The bending load and bending moment (*M*) in the leaf present a decreasing tendency from the base to the apex. This occurs because a random unit of the leaf must support the weight and loading of the portion of the leaf beyond it. The units near the base must resist a greater load, and hence bending moment, than those near the apex [16]. There are more fiber bundles, vascular bundles, diaphragms, and foam tissues existing in the base than that in the apex, which makes the bending stiffness decreased from the base to the apex.

Table 1 reports section modulus in bending and the second moment of area for the leaf specimen. The bending strength (*σ*), flexural modulus (*E*), and the surface elongation at break (*ε*) are computed by (4), (5), and (6), respectively. The variations in flexural strength, flexural modulus, and elongation at break of three *Typha* leaves from the base to the apex are plotted in Figures 4(b), 4(c), and 4(d), respectively. The flexural strength and flexural modulus of *Typha* leaves vary from 0.71 MPa to 7.12 MPa and from 2.16 MPa to 269.73 MPa, respectively. The values of flexural strength and flexural modulus of flat number 3 are the highest, and the values of the convex number 2 are the lowest. Figures 4(b) and 4(c) show the flexural strength and flexural modulus that increase slowly from the base to the middle of leaves and then significantly increase from the middle to the apex of leaves. The variation tendency may relate to the size changes of leaf cross sections. Both the section modulus in bending and second moment of area sharply decrease due to the rapid reduction in the cross-sectional area near the leaf apex, leading to the significant increase of the flexural strength and flexural modulus.  Figure 4(d) illustrates the surface elongation at break on the base of the leaf (38.62%), which is much higher than that at the apex (2.21%). The surface elongation at break of concave number 1 is higher than flat number 3, and convex number 2 is the lowest. It can be concluded that the base of the leaf blade with a maximal second moment of the area can with stand a greater moment. The apex of the leaf blade has higher flexural strength and flexural modulus, which makes it more difficult to bend. And the maximal surface elongation at break of the leaf base will resist bending deformation effectively. Therefore, the *Typha* leaf can prevent buckling derived from nature.

## 4. Conclusion

In this paper, we investigated the macroscopic morphology, microstructure, and the flexural properties of the *Typha* leaves. The results can be summarized as follows.

1. The *Typha* leaf is a lightweight cantilever beam, consisting of epidermis, diaphragm, partition, nonlignified fiber cables, and foam tissues. The total porosity of the leaf blade is approximately 96%, exhibiting the characteristics of high strength and low density.
2. The cross section of the leaf varies obviously from crescent to less concave shape, and the leaf is finally biconvex in its distal portion. The cross-sectional shape performance has been examined with shape transformers. The high *ψ_I_/ψ_A_* ratio of the cross-sectional shape demonstrates that the *Typha* leaf has a quite efficient flexural stiffness.
3. The bending properties of the *Typha* leaf are affected by the factors, such as the development level of the epidermal tissue, the vascular bundle, the mechanical tissue, and the shape and size of the cross section of the leaf blade. The bending load and bending moment in the leaf show a decreasing tendency from the base to the apex of leaves. However, both the flexural strength and the flexural modulus significantly increase from the base to the apex, and the values of flat number 3 are the highest. The surface elongation at break on the base of the leaf is much higher than that of the apex, and the value of convex number 2 is lowest.
4. The present study provides us more information about the structure and mechanical properties of the *Typha* leaf and may be useful for fabrication and design of engineering new bionics lightweight structures.

## Supplementary Material

Figure In-situ three-point bending test apparatus with maximum load 50 N.

## Figures and Tables

**Table 1:** Geometry of cross sections of the *Typha* leaf specimens.

Specimen number	Leaf cross section	*B* (mm)	Area (mm^2^)	*ψ_A_*	*I* (mm^4^)	*W* (mm^3^)	*ψ_I_*
		*H* (mm)					
1-1	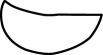	15.42	83.01	0.70	328.43	64.15	0.56
		7.71					
1-2	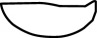	14.88	62.95	0.73	142.57	43.77	0.59
		5.81					
1-3	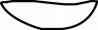	14.77	49.57	0.69	75.92	27.67	0.54
		4.86					
1-4	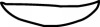	15.13	38.06	0.63	34.91	16.31	0.44
		3.99					
1-5	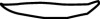	14.94	27.06	0.55	10.87	7.39	0.24
		3.32					
1-6	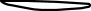	13.76	14.14	0.70	2.16	2.4	0.59
		1.47					
2-1	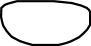	14.09	81.82	0.81	270.54	71.28	0.63
		7.15					
2-2	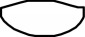	12.99	58.31	0.75	118.51	38.86	0.52
		5.95					
2-3	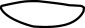	13.03	40.42	0.69	46.89	20	0.49
		4.46					
2-4	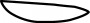	13.91	31.52	0.69	24.71	12.55	0.61
		3.27					
2-5	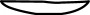	13.84	22.73	0.63	8.5	6.47	0.42
		2.59					
2-6	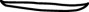	13.69	14.10	0.57	2.36	2.16	0.35
		1.81					
3-1	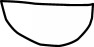	14.42	74.63	0.75	233.8	61.12	0.59
		6.91					
3-2	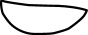	13.42	48.69	0.73	80.74	29.41	0.59
		4.96					
3-3	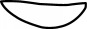	13.33	37.81	0.66	44.23	18.21	0.51
		4.27					
3-4	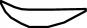	13.47	27.58	0.54	20.24	8.2	0.33
		3.79					
3-5	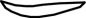	13.35	19.27	0.50	6.49	4.66	0.24
		2.89					
3-6	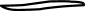	13.24	11.39	0.54	1.71	1.98	0.37
		1.61					
