# Supplementary figures and images for: The Structure and Flexural Properties of Typha Leaves
Source: Appl Bionics Biomech. 2017 Oct 15;2017:1249870. doi: 10.1155/2017/1249870 (PMC5662842; doi:10.1155/2017/1249870)

**
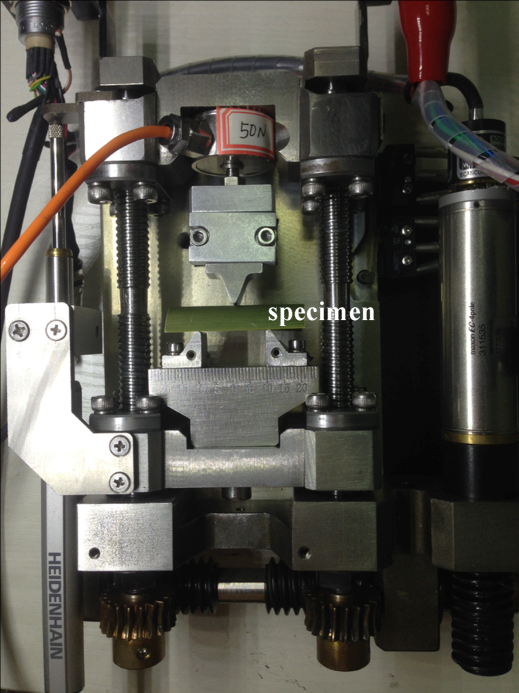
**

FIGURE: In-situ three-point bending test apparatus with maximum load 50 N.

Supplement: Supplementary file 1 — Figure In-situ three-point bending test apparatus with maximum load 50 N. [file 1249870.f1.docx]
